# SARS-like Coronaviruses in Horseshoe Bats (*Rhinolophus* spp.) in Russia, 2020

**DOI:** 10.3390/v14010113

**Published:** 2022-01-09

**Authors:** Sergey Alkhovsky, Sergey Lenshin, Alexey Romashin, Tatyana Vishnevskaya, Oleg Vyshemirsky, Yulia Bulycheva, Dmitry Lvov, Asya Gitelman

**Affiliations:** 1 D.I. Ivanovsky Institute of Virology of N.F. Gamleya National Center for Epidemiology and Microbiology of Ministry of Health of Russian Federation, 18 Gamaleya Street, 123098 Moscow, Russia; t_vish77@mail.ru (T.V.); boulychevayuli@ya.ru (Y.B.); dk_lvov@mail.ru (D.L.); gitelman_ak@mail.ru (A.G.); 2 Reference Center on Coronavirus Infection of N.F. Gamleya National Center for Epidemiology and Microbiology of Ministry of Health of Russian Federation, 18 Gamaleya Street, 123098 Moscow, Russia; 3 Scientific Research Institute of Medical Primatology of Russian Academy of Science, 177 Mira Street, Veseoloe Village, 354376 Sochi, Russia; lenshin-s@mail.ru (S.L.); olegvyshem@gmail.com (O.V.); 4 Federal State Budgetary Institution Sochi National Park of Ministry of Natural Resources and Environment of Russian Federation, 74 Kurortniy Avenue, 354002 Sochi, Russia; romashin@sochi.com

**Keywords:** SARS-CoV, SARS-CoV-2, bat SARS-like coronaviruses, SARS-CoV-like viruses, viral metagenomics, coronavirus, horseshoe bats, zoonotic viruses, *Rhinolophus*

## Abstract

We found and genetically described two novel SARS-like coronaviruses in feces and oral swabs of the greater (*R. ferrumequinum*) and the lesser (*R. hipposideros*) horseshoe bats in southern regions of Russia. The viruses, named Khosta-1 and Khosta-2, together with related viruses from Bulgaria and Kenya, form a separate phylogenetic lineage. We found evidence of recombination events in the evolutionary history of Khosta-1, which involved the acquisition of the structural proteins S, E, and M, as well as the nonstructural genes ORF3, ORF6, ORF7a, and ORF7b, from a virus that is related to the Kenyan isolate BtKY72. The examination of bats by RT-PCR revealed that 62.5% of the greater horseshoe bats in one of the caves were positive for Khosta-1 virus, while its overall prevalence was 14%. The prevalence of Khosta-2 was 1.75%. Our results show that SARS-like coronaviruses circulate in horseshoe bats in the region, and we provide new data on their genetic diversity.

## 1. Introduction

Horseshoe bats (Rhinolophidae: *Rhinolophus*) are considered a main natural reservoir and source of zoonotic coronaviruses (CoV), which caused epidemic outbreaks of severe acute respiratory syndrome (SARS) and the COVID-19 pandemic in 2002 and 2019, respectively [1,2]. These viruses, designated SARS-CoV and SARS-CoV-2, together with related viruses found in bats and other animals (SARS-like coronaviruses or SARS-CoV-like viruses), belong to the subgenus *Sarbecovirus* of the genus *Betacoronavirus* of the family *Coronaviridae* [3]. Horseshoe bats are widely distributed in Asia, Europe, and North Africa. In East Asia (in particular, China), SARS-CoV-like viruses circulate in multiple rhinolophid species; however, the Chinese rufous (*R. sinicus*) and the greater (*R. ferrumequinum*), intermediate (*R. affinis*), Malayan (*R. malayanus*), the least (*R. pusillus*), and king (*R. rex*) horseshoe bats seem to be of major importance [4]. In Europe, SARS-CoV-like viruses were found in the greater, the lesser (*R. hipposideros*), the Mediterranean (*R. euryale*), Mehely’s (*R. mehelyi*), and Blasius’ (*R. blasii)* horseshoe bats [5,6,7,8]. The prevalence of SARS-like coronaviruses among bats in different caves/colonies can vary from 0% to 60% [4,7,9,10]. In Russia, three species of horseshoe bats (the greater, lesser, and Mediterranean) are common in the southern regions, lying below about 44° north latitude, mostly including North Caucasus and Crimea. In the present work, we hypothesized that SARS-like coronaviruses circulate in the region in local populations of horseshoe bats. To test this hypothesis, we examined the colonies of bats located in the southern macroslope of the Greater Caucasus on the northern coast of the Black Sea in Russia. Using metagenomic analysis, we found and genetically described two new SARS-like coronaviruses in feces and oral swabs of the greater and lesser horseshoe bats. Further PCR analysis showed a high degree of infection of bats with discovered viruses in some locations.

## 2. Materials and Methods

### 2.1. Sample Collection

**Figure 1:**
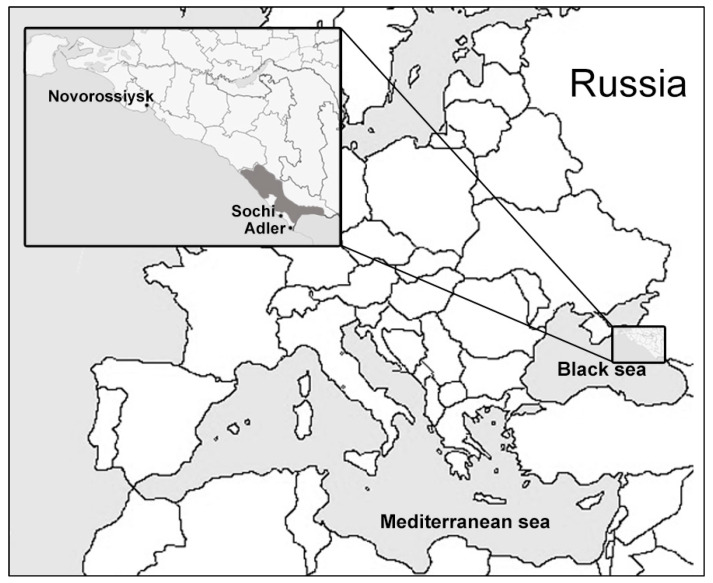
Map of the region where bat samples were collected. The location of Sochi National Park and the surrounding area is shown in gray.

The samples from bats were collected in Sochi National Park (Sochi-Adler, Krasnodar krai, Russia) and surrounding areas in March–October 2020. The Sochi National Park is located on the southern macroslope of the Greater Caucasus, descending to the northern coast of the Black Sea (Figure 1). The park keeps records of more than 300 karst formations (caves, breaks, mines, clefts, etc.) that are natural refuges for bats and other troglophilous animal species. Bats were caught by hand in eight locations including five caves, as well as the basements and attics of houses (Table 1). The bats were caught in the frame of the ongoing surveillance of bat populations constantly carried out in the park. The collection of samples was approved by the Scientific Council and the Ethics Committee of Sochi National Park. The species of the animals were determined on the basis of their morphological characteristics by an experienced park zoologist. The length of the forearm and weight of the animals were measured. To collect bat oral swabs (saliva and buccal cells), a urological swab was placed in the bat’s mouth for 10–15 s and then placed in 250 μL of phosphate-buffered saline (PBS). To collect feces, an animal was placed in an individual small white cotton bag for 10–15 min. After the animal was released, the feces were collected from the walls of the bag into cryovials. No bats were harmed during sample collection. A total of 120 samples of oral swabs and 77 samples of feces from five species of bats were collected (Table 1). The samples were delivered to the laboratory on ice and stored at −70 °C until analysis.

### 2.2. Metagenomic Analysis

For metagenomic analysis, the feces were suspended and homogenized in 0.5 mL of PBS and pooled to 0.1 mL in three samples (there were feces from 20–30 animals in the pool). The pooled samples were clarified by centrifugation (10,000× *g*, 15 min) and treated with DNase I and RNase I_f_ (NEB, Hitchin, Great Britain) for the removal of naked non-capsid nucleic acid. The viral particles were sedimented from the treated samples by ultracentrifugation (30,000× *g*, 1 h) through 2 mL of 20% sucrose. Virus plaque was resuspended in 0.5 mL of PBS. Total RNA was isolated from 0.25 mL of obtained solution with TRIzol LS reagent (Thermo Fisher Scientific, Waltham, MA, USA).

Total RNA from oral swabs was isolated with TRIzol LS reagent from individual samples and pooled to 20 µL in five pooled samples (20–25 samples in the pool). The pooled RNA was precipitated using isopropyl alcohol with the addition of glycogen, followed by additional clarification with an RNeasy MinElute Cleanup kit (Qiagen, Hilden, Germany). An NEBNext rRNA Depletion kit (NEB, Hitchin, Great Britain) was used to remove bacterial and eukaryotic rRNA from the total RNA isolated from the pooled samples. The treated RNA was used for cDNA library preparation with a NEBNext Ultra II RNA library kit for Illumina (NEB, Hitchin, Great Britain).

The libraries were sequenced on a HiSeq4000 instrument (Illumina, San Diego, CA, USA) at the Resource Center “BioBank” of the Research Park of Saint Petersburg State University (Saint-Petersburg, Russia). The reads were filtered by quality, trimmed to remove the adapter sequences, and assembled de novo using the CLC Genomics Workbench 7.0 software (Qiagen, Hilden, Germany). The obtained contig sequences were analyzed using the blastx algorithm using the DIAMOND v. 2.0.13 software (https://github.com/bbuchfink/diamond) (accessed on 14 November 2021) [11] against the nr “Viruses” database that included all the reference viral sequences available in GenBank as of December 2020.

### 2.3. Genetic and Phylogenetic Analysis

The nucleotide and deduced amino-acid sequences were aligned using ClustalW implemented in the MEGAX v. 10.2 software (https://www.megasoftware.net) (accessed on 14 November 2021) [12]. In total, 40 complete genome sequences of certain sarbecoviruses from GenBank were used for the analysis. The alignments obtained for the ORF1a, ORF1b, S, ORF3, E, M, ORF6, ORF7a, ORF7b, and N proteins were used to determine value of identity (%) between different viruses. For phylogenetic analysis the best substitution model was evaluated for each alignment using the model selection module in the MEGAX software. Phylogenetic trees for RdRp, S, and N gene were inferred using the “maximum likelihood” method using the appropriate model (GTR + G + I) with 1000 bootstrap replicates using the MEGAX software. Similarity plot analysis was conducted using the SimPlot v. 3.5.1 software (https://sray.med.som.jhmi.edu/SCRoftware/SimPlot) (accessed on 14 November 2021) [13]. Possible recombination was analyzed using the RDP5 v. Beta 5.05 software (http://web.cbio.uct.ac.za/~darren/rdp.html) (accessed on 14 November 2021) [14]. RDP, GENECONV, bootscan, maximum chi-square, Chimaera, 3Seq, and SiScan methods were used with default setting and Bonferroni corrected *p* value cutoff of 0.05 as implemented in RDP5 v. Beta 5.05 software. Recombination events were accepted if detected by at least four methods and confirmed by phylogenetic analysis.

### 2.4. RT-PCR Analysis

Primers and probes for the specific detection of discovered coronaviruses (Kh1_pr FAM-ACCTGTGCCTGTGAGTCCATT-BQ1, Kh1_F CACTGTTGGTGTAGGTTAC, and Kh1_R CTGGAATGACTGAAATCTCTTA for Khosta-1; Kh2_pr HEX-AAGCACACCAACGACACCAGCATCTC-BQ2, Kh2_F CGCCAAGCACTATTAAAGACAG, and Kh2_R CGAAGTCGTACCAGTTTCCA for Khosta-2) were developed on the basis of obtained sequences using the Beacon 7.0 software (Premier Biosoft, San Francisco, CA, USA). Real-time RT-PCR was conducted with the TaqPath 1-Step Multiplex Master Mix (Thermo Fisher Scientific, Waltham, MA, USA) and total RNA isolated using TRIzol LS reagent from individual oral swabs and fecal samples. Briefly, 5 μL of isolated RNA was added to a 15 μL reaction mixture containing 1X TaqPath 1-Step Multiplex Master Mix, 400 nM of forward and reverse primers, and 200 nM of corresponding probe. The total volume of the PCR mixture was 20 μL. RT-PCR analysis was performed on CFX96 real-time PCR detection system (Bio-Rad, Hercules, CA, USA). The thermal cycling profile consisted of incubation steps at 50 °C for 30 min for reverse transcription and incubation 30 s at 95 °C for Taq-polymerase activation followed by 45 cycles of 10 s at 95 °C and 30 s at 55 °C. 

The species *R. hipposideros* and *R. ferrumequinum* were confirmed by the partial sequencing of the mitochondrial cytochrome b gene of the tested samples. For other bats, the genetic confirmation of species was not carried out.

## 3. Results

### 3.1. Results of Sequencing of the Samples

In total, 124,522,978 reads for three pooled fecal samples and 170,112,341 reads for five pooled oral swab samples were obtained. The reads were de novo assembled in contigs and analyzed using the blastx algorithm for the presence of viral sequences. The search results revealed two extended contigs with a length of approximately 29 kb with open reading frames (ORFs) similar to members of the genus *Betacoronavirus* in Pools 1 and 3 of the fecal samples. Following further analysis, a near-complete genome of two novel SARS-like coronaviruses was sequenced. Matching contigs were also found in corresponding oral swab samples, but with smaller length and coverage. The two discovered SARS-like coronaviruses were named BtCoV/Khosta-1/Rh/Russia/2020 and BtCoV/Khosta-2/Rh/Russia/2020 and placed in GenBank under accession numbers MZ190137 and MZ190138, respectively. They are herein referred to as Khosta-1 and Khosta-2, respectively.

### 3.2. Genetic and Phylogenetic Analysis

**Figure 2:**
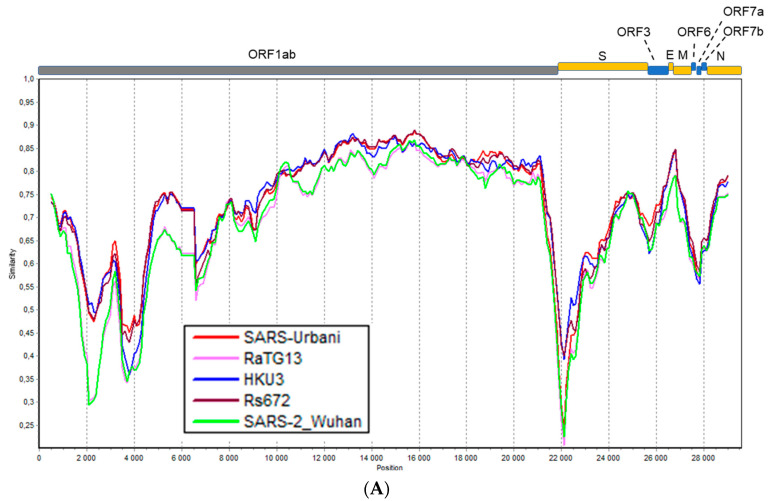

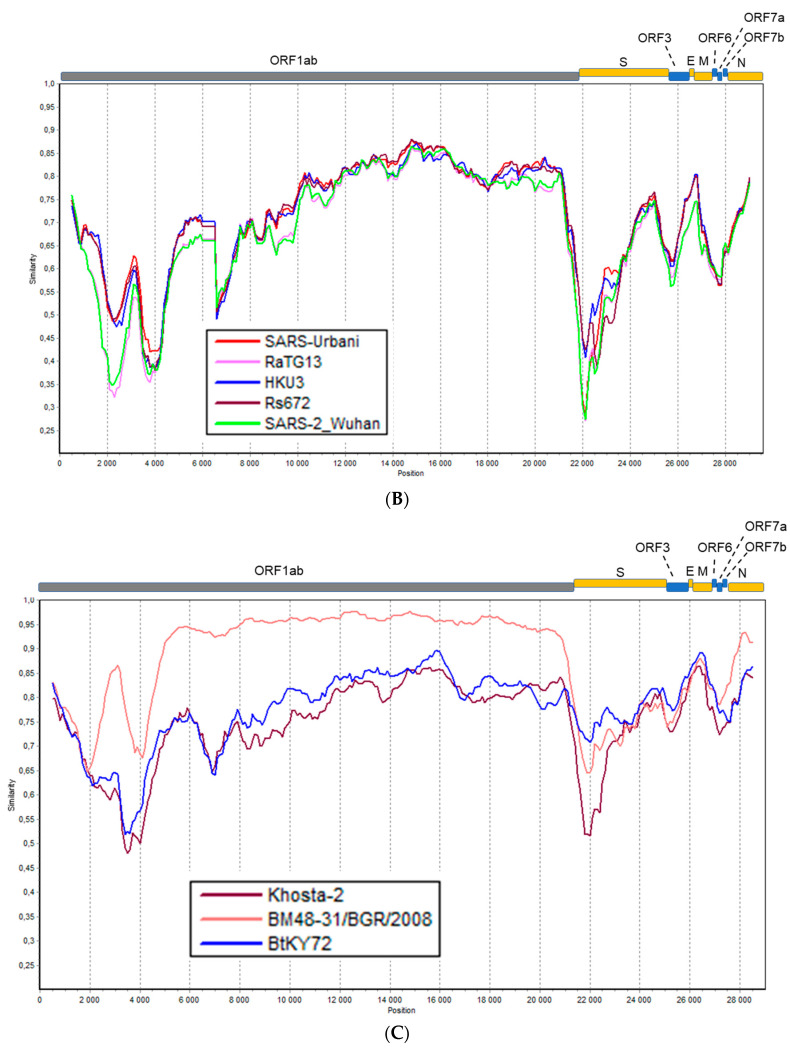

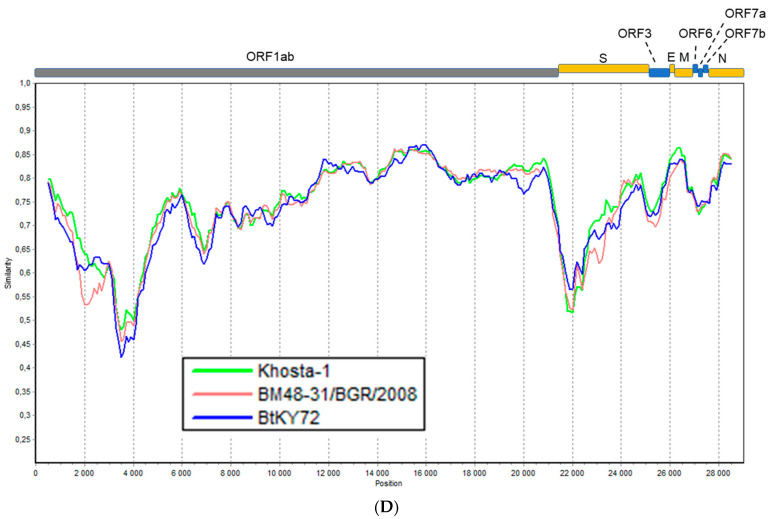
Simplot analysis of Khosta-1 and Khosta-2 with SARS-CoV, SARS-CoV-2, and related viruses. RaTG13, HKU3, and Rs672 were used as representatives of bat SARS-CoV-like viruses from Asia. (**A**) Khosta-1 was used as a query sequence, and SARS-CoV, RaTG13, HKU3, Rs672, and SARS-CoV-2 were used as reference sequences. (**B**) Khosta-2 was used as a query sequence, and SARS-CoV, RaTG13, HKU3, Rs672, and SARS-CoV-2 were used as reference sequences. The sequences of the ORF8 gene, which is absent in Khosta-1 and Khosta-2, were removed from alignment before analysis. (**C**) Khosta-1 was used as a query sequence, and Khosta-2, BM48-31/BGR/2008, and BtKy72 viruses were used as reference sequences. (**D**) Khosta-2 was used as a query sequence, and Khosta-1, BM48-31/BGR/2008, and BtKY72 were used as reference sequences. The analysis was performed using the Kimura (two-parameter) model, with a window size of 1000 bases and a step size of 100 bases.

The genomic organization of Khosta-1 and Khosta-2 was similar to that of other SARS-like coronaviruses (Figure 2). Approximately two-thirds of the genome of coronaviruses is occupied by ORF1a and ORF1b genes, which encode the proteins of the replicative complex and are translated as the ORF1ab polyprotein due to ribosomal shifting. The remainder of the genome contains genes of structural proteins (S, E, M, and N), which form a virion, as well as several nonstructural proteins (ORF3, ORF6, ORF7, ORF8, ORF9, and ORFX), the presence and structure of which vary in different viruses [3]. The genome organization of Khosta-1 and Khosta-2 had the greatest similarity to the BtCoV/BM48-31/2008 and BtKY72 viruses—two SARS-like coronaviruses found in horseshoe bats in Bulgaria and Kenya in 2008 and 2007, respectively [8,15]. The common peculiarity of Khosta-1, Khosta-2, BtCoV/BM48-31/2008, and BtKY72 is the absence of the ORF8 gene, which is common in bat SARS-like coronaviruses from East and Southeast Asia.

Khosta-1 and Khosta-2 showed 76–78.2% nt identities with SARS-CoV, SARS-CoV-2, and related viruses found in China according to a full-length genome comparison. The full-length genome of Khosta-1 was most similar to that of the European strain BtCoV/BM48-31/2008 (89.5% nt identity) and had a lower level of similarity to BtKY72 (81.7% nt). The Khosta-2 genome, by contrast, had a near-identical similarity to BtCoV/BM48-31/2008 and BtKY72 (both 79.8% nt), as well as strains isolated in East and Southeast Asia. The genome sequence similarity of Khosta-1, Khosta-2, and other sarbecoviruses was analyzed using the Simplot software (Figure 2). In general, the genetic similarity of Khosta-1 and Khosta-2 with Eastern strains did not exceed 85% nt identity in the most conserved part of the coronavirus genome, i.e., the ORF1b gene, with a decrease to 20–30% in variable regions (Figure 2A,B). The analysis showed the highest degree of similarity of Khosta-1 to BtCoV/BM48-31/2008 in the ORF1ab and N genes, as well as a decrease in the similarity in the S-ORF7b region (Figure 2C,D).

Pairwise alignments of the deduced proteins of Khosta-1 and Khosta-2 virus with those of other SARS-like coronaviruses also showed the highest identity with BtCoV/BM48-31/2008 and BtKY72 (Table 2). Khosta-1 was most closely related to BtCoV/BM48-31/2008, with 92.5% aa and 99% aa identity in the conservative ORF1a and ORF1b proteins, respectively. The similarity of Khosta-1 to SARS-CoV and related viruses from China was, on average, 81.5% aa identity in the ORF1a protein and 96% aa identity in the ORF1b protein. A comparison of Khosta-1 with SARS-CoV-2 viruses revealed 77.5% and 94.2% aa identity in the ORF1a and ORF1b proteins, respectively. Despite the high similarity of Khosta-1 and BtCoV/BM48-31/2008 in the ORF1a and ORF1b proteins, the structural proteins S, E, and M of Khosta-1 were more similar to those of the Kenyan virus BtKY72. Khosta-1 and BtKY72 shared 89.1%, 98.7%, and 97.29% aa identity for the S, E, and M proteins, whereas these values for Khosta-1 and BtCoV/BM48-31/2008 were 84.37%, 89.47%, and 95%, respectively. The N protein of Khosta-1 was more similar to that of BtCoV/BM48-31/2008 (96.64% aa identity) than BtKY72 (92.6% aa identity).

By contrast, Khosta-2 did not exhibit such an increased similarity with some groups of sarbecoviruses and had 79–81% aa identity with SARS-CoV viruses and 76–77% aa identity with SARS-CoV-2 and related viruses in the ORF1a protein. The ORF1b protein of Khosta-2 had 93.5–95% aa identities with all the other bat SARS-like coronaviruses. A comparison of the proteins of Khosta-1 and Khosta-2 showed that these viruses differ from each other at about the same level at which Khosta-2 differs from other bat SARS-like coronaviruses (Table 2).

### 3.3. Phylogenetic Analysis

**Figure 3:**
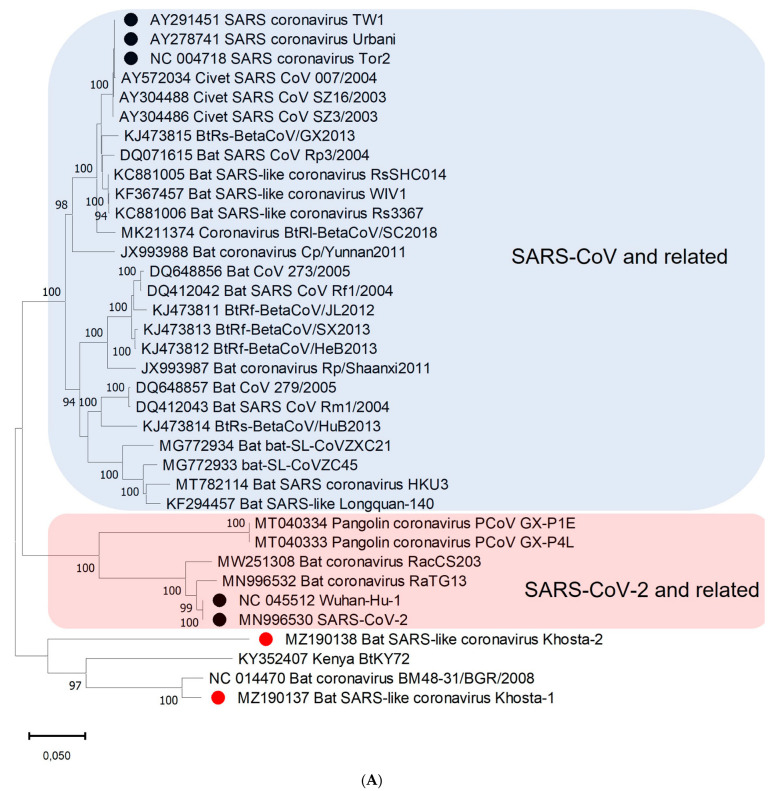

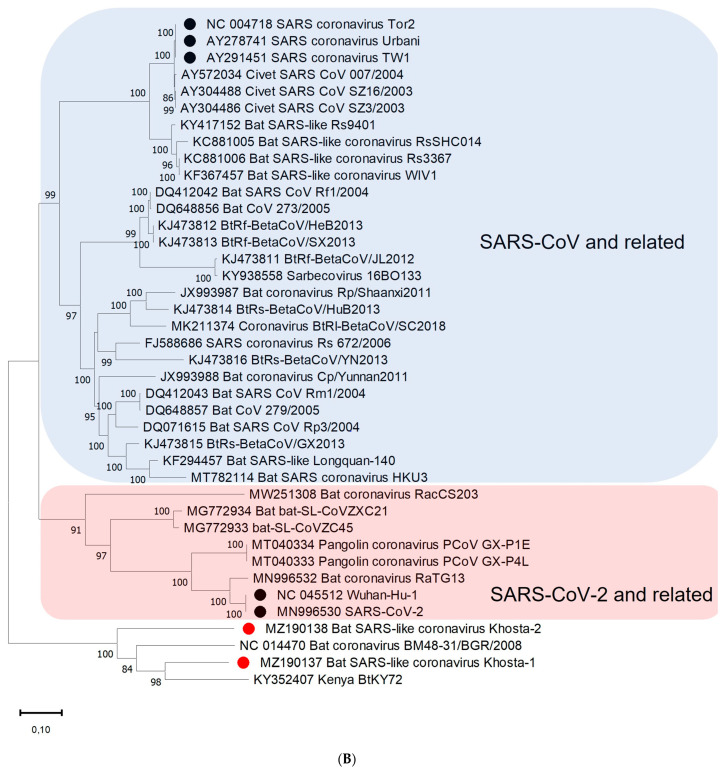

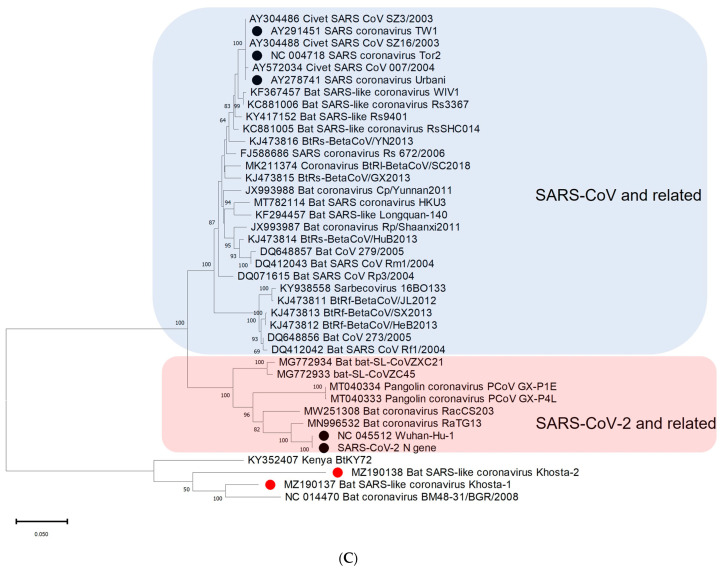
Phylogenetic trees inferred using maximum likelihood method according to an analysis of nucleotide sequences of the RdRp gene (2766 nt) (**A**), nucleotide sequences of the S gene (3822 nt (SARS-CoV-2 numbering)) (**B**), and nucleotide sequences of N gene (1257 nt) (**C**) of certain sarbecoviruses. The percentage of trees in which the associated taxa clustered together is shown next to the branches (values higher 50% are shown). SARS-CoV and SARS-CoV-2 are marked by black circles; Khosta-1 and Khosta-2, described in the present work, are marked by red circles. The trees were inferred using GTR + G + I model with 1000 bootstrap replicates using the MEGAX.

A phylogenetic analysis based on the nucleotide sequences of the conserved RdRp gene showed that Khosta-1, Khosta-2, BtCoV/BM48-31/2008, and BtKY72 form a monophyletic lineage located outside the SARS-CoV and SARS-CoV-2 lineages of the *Sarbecovirus* subgenus (Figure 3A). A separate cluster of this group of viruses was also formed in the phylogenetic tree for the S gene (Figure 3B) and the N gene (Figure 3C). The topology of the trees confirmed a probable recombination event in the evolutionary history of Khosta-1. In the RdRp and N trees, Khosta-1 was grouped together with BtCoV/BM48-31/2008, whereas, in the S gene tree, it was grouped together with BtKY72. 

### 3.4. Recombination Analysis

**Figure 4:**
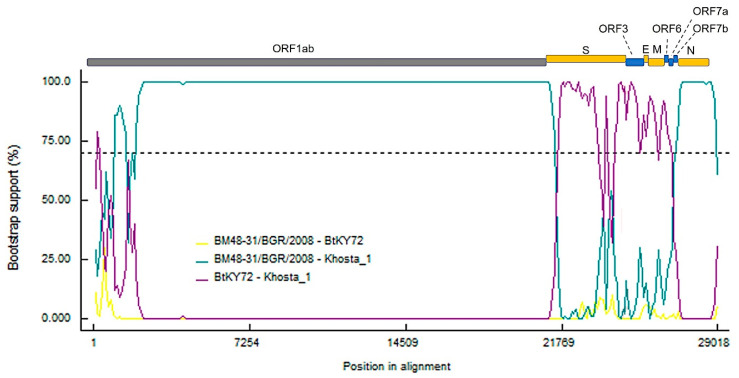
Results of bootscan analysis of recombination events in Khosta-1 genome using RDP5 software. The analysis was performed using the Jukes and Cantor (1969) model, with a window size of 1000 bases, a step size of 100 bases and a number of bootstrep replicates of 100 as implemented in RDP5 program. Cutoff percentage (70%) shown by dashed line. The colors of the lines correspond to different pairs of analyzed viruses as indicated in the legend.

The results of analysis carried out using the RDP5 software by implementing different methods in the program showed clear signals of recombination events in the evolutionary history of Khosta-1. The program identified three recombination events localized in the S-ORF7b region, detected by four to six different methods and confirmed by phylogenetic analysis. Of these, the event involving the S gene region is strictly supported by six different methods: RDP (*p* value 1.982 × 10^−81^), GENECONV (*p* value 2.6 × 10^−03^), BootScan (*p* value 7.973 × 10^−24^), MaxChi (*p* value 4.343 × 10^−34^), Chimeara (*p* value 2.698 × 10^−27^), SiScan (*p* value 4.928 × 10^−12^), 3Seq (*p* value 4E-30 × 10^−300^). Figure 4 presents the results of a bootscan analysis, clearly showing recombination events presumably included the acquisition of the S-ORF7b region in an ancestor of the Khosta-1 virus from a virus closely related to BtKY72.

### 3.5. Analysis of Receptor-Binding Motif (RBM) and S1/S2 Cleavage Site of S Protein

**Figure 5:**
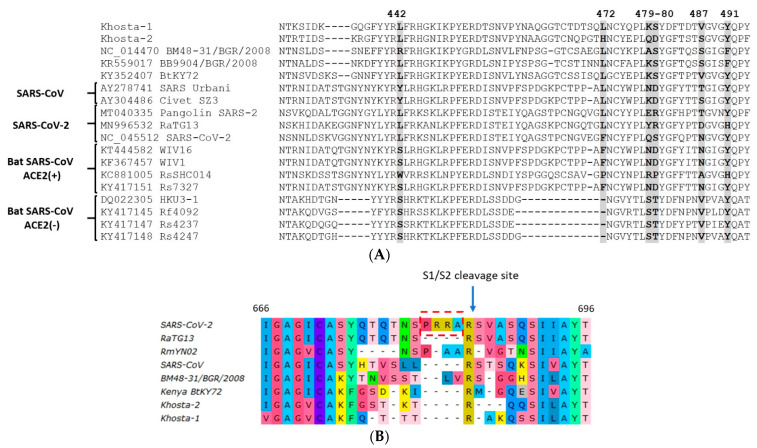
Amino acid alignment of the receptor-binding motif (RBM) of the receptor-binding domain (RBD) of the S protein of Khosta-1 and Khosta-2 of certain sarbecoviruses. Five positions (442, 472, 479, 480, and 487; SARS-CoV Urbani numbering) in the RBM that are thought to be important for adaptation of SARS-CoV-like viruses to the human ACE2 receptor [17] are shown in bold. Bat SARS-CoV-like viruses that are capable or incapable of utilizing the ACE2 receptor are marked with “ACE2(+)” or “ACE2(−)”, respectively (**A**). Amino acid alignment of the region around the S1/S2 cleavage site in SARS-CoV-2, SARS-CoV, Khosta-1, Khosta-2, and certain bat SARS-CoV-like viruses. Four-amino acids insertion (PRRA) that forms polybasic furin cleavage site in SARS-CoV-2 is highlighted with a red border (**B**).

An alignment of the amino-acid sequences of the RBM of Khosta-1 and Khosta-2 with certain sarbecoviruses is presented in Figure 5. This is a highly variable region where multiple substitutions and deletions occur among SARS-CoV-related viruses. Khosta-1 and Khosta-2, as well as BtCoV/BM48-31/2008, had a common deletion of four aa in the N-terminal region of the RBM. This deletion partially overlapped with a deletion characteristic of HKU3 and related strains of bat SARS-CoV-like viruses that are unable to bind the angiotensin-converting enzyme 2 (ACE2) receptor. We analyzed the aa positions in the RBM that are thought to be crucial for the binding of the ACE2 receptor and, therefore, important for the adaptation of bat SARS-like coronaviruses to human transmission [16,17]. Khosta-1 and Khosta-2 shared a common amino acid (L) only at position 442, which is also inherent in SARS-CoV-2 and related viruses. Despite the significant genetic distance between Khosta-1 and BtKY72, crucial positions in the RBM and their nearby amino acids coincided. By contrast, positions 479, 480, and 487 of Khosta-2 coincided poorly with other groups of viruses (Figure 5A).

The notable feature of SARS-CoV-2 is a four-amino acid insertion (RRRA) at the junction of S1 and S2 subunits of the spike protein. This insertion forms polybasic cleavage site for furin proteases and probably has a role in determining viral infectivity and pathogenicity [18]. Analysis of this region showed that Khosta-1 and Khosta-2, similar to other bat SARS-CoV-like viruses, do not have insertions in this region that could form an additional site for furin protease (Figure 5B).

### 3.6. PCR Testing

We developed primers and probes for the specific detection of Khosta-1 and Khosta-2 viruses in feces and oral swabs by real time RT-PCR. The results of the PCR testing of the samples are presented in Table 1. The RNA of Khosta-1 was detected mostly in the greater horseshoe bats collected in Kolokolnaya cave. All four cases revealed positive oral swabs belonging to animals with positive fecal samples. In other locations, Khosta-1 virus was detected only in two fecal samples—from the greater horseshoe bat from Khosta 1 cave and the lesser horseshoe bat from Partizanskaya cave. Furthermore, the RNA of Khosta-1 was detected in feces much more often than in oral swabs, as well as at a higher viral load (according to the Ct values; data not shown). The RNA of Khosta-2 virus was detected in two lesser horseshoe bats collected in the basement of a building at the Research Institute of Medical Primatology. In one animal, the RNA of Khosta-2 was detected in both feces and oral swabs, whereas, in another, it was only detected in the oral swab.

## 4. Discussion

Increasing pieces of evidence from multiple studies do suggest an immediate ancestors of SARS-CoV and SARS-CoV-2 most likely originated from viruses circulated in different species of horseshoe bats [2,19,20,21,22,23]. To date, bat viruses closest related to SARS-CoV-2 have been found in *R. affinis* (strain RaTG13), *R. malayanus* (strain RmYN02), and *R. pusillus* (strain RpYN06) collected in Yunnan province of China [19,23,24]. Other strains, more distant or exhibited high sequence identity to SARS-CoV-2 in certain regions of the genome, were found in bats in Zhejiang province of China (strains ZXC21 and ZC45 from *R. pusillus*), Thailand (strain RacCS203 from *R.* *acuminatus*), Cambodia (strains RshSTT182 and RshSTT200 from *R.* shameli), Laos (strains BANAL-52, -103, -236 from *R. malayanus*, *R. pusillus,* and *R. marshalli*, respectively), and Japan (strain Rc0319 from *R. cornutus*) [25,26,27,28,29]. Genetic and phylogenetic analysis carried out in these and other studies shows that the genome of sarbecoviruses is subject to frequent recombinations and genome of SARS-CoV-2, similar to related viruses, probably has a complex mosaic origin. Horseshoe bats are widespread and, presumably, SARS-like coronaviruses circulate across the regions of their distribution, including Asia, Europe, and North Africa. However, little information exists on the genetic diversity of bat SARS-like coronaviruses in regions outside East and Southeast Asia. We described here two novel SARS-like coronaviruses circulating in horseshoe bats in the southern region of Russia. Khosta-1 and Khosta-2 viruses are closely related to viruses recently described in Bulgaria (strain BtCoV/BM48-31/2008) and Kenya (strain BtKY72) [8,15]. Together, they form a separate “western” (as they are found to the west of regions home to horseshoe bats) phylogenetic lineage of bat SARS-like coronaviruses. A feature of these viruses is the absence of the ORF8 gene, which is common in SARS-CoV, SARS-CoV-2, and most bat SARS-like coronaviruses of eastern lineages.

SARS-CoV and SARS-CoV-2 recognize the host’s angiotensin-converting enzyme 2 (ACE2) as their receptor. Amino acids (442, 487, 479, 487, and 491) crucial for binding of ACE2 are located in the RBM of the S protein [17,30,31,32,33]. These amino acids and their surrounding residues in the RBM of Khosta-1 and Khosta-2, as in most other bat SARS-like coronaviruses, are quite different from those in SARS-CoV and SARS-CoV-2. Most bat SARS-like coronaviruses are unable to bind the ACE2 receptor of humans and, thus, are not infectious toward their cells [34]. However, several strains of bat SARS-like coronaviruses that can use the ACE2 receptor have been recently found in the Chinese rufous (*R. sinicus*) and the intermediate (*R. affinis*) horseshoe bats in China [35,36,37,38]. Distantly related to SARS-CoV-2 strains (RsYN04, RmYN05 and RmYN08, all also from China) have been found to bind to the human ACE2 receptor, albeit with very low affinity [23]. Finally, strains with an almost identical to SARS-CoV-2 RBD and a high receptor binding capacity have been found in bats in Laos [28]. These data suggest that the ability to bind a human ACE2 receptor could arise naturally and independently in different phylogenetic lineages of sarbecoviruses.

Since recombination is of great importance in the evolution of coronaviruses, we analyzed possible recombination events in the western lineage of bat SARS-like coronaviruses. Despite the small number of known full-length sequences (only four), we observed evidence of recombination in the evolutionary history of Khosta-1. The alleged recombination event involved the acquisition of structural proteins S, E, and M, as well as nonstructural genes ORF3, ORF6, ORF7a, and ORF7b, from a virus that is closer to the Kenyan isolate BtKY72 than to the European strain BtCoV/BM48-31/2008. Accordingly, we can assume that the genetic diversity of viruses in the region is significantly higher than currently established, and there is a constant exchange of genes across viruses. These findings require further investigation of the diversity of circulating variants, with particular emphasis on the diversity of the S gene.

Using RT-PCR, we showed that 14% of tested horseshoe bats were positive for Khosta-1 virus and 1.75% were positive for Khosta-2 virus. However, most of the Khosta-1-positive samples were found in only one cave (Kolokolnaya cave), where the infection rate of greater horseshoe bats reached 62.5%. This bias, together with the small number of samples from other locations, makes it difficult to accurately estimate the prevalence of Khosta-1 in the region; hence, further research is required. The closest European region where such studies have been carried out is Bulgaria; according to the data obtained by Drexler et al. (2010), SARS-like coronaviruses were detected in 13.3% of greater horseshoe, 15.9% of Blasius’s horseshoe, 30.8% of Mehely’s horseshoe bat, and 32.1% of Mediterranean horseshoe bats [8]. Other studies found 38.8% positivity in lesser horseshoe bats in Slovenia and 37.9% positivity in greater horseshoe bats in France [5,6]. All these data show that the prevalence of SARS-like coronaviruses in horseshoe bats in Europe can vary widely across different species, locations, and possibly the time of year of observation.

In conclusion, we showed that SARS-like coronaviruses circulate in horseshoe bats in the southern region of Russia, and we provided new information on their genomic diversity. The genetic diversity, prevalence, host range, and potential threat to humans of these viruses remain to be determined.

## Figures and Tables

**Table 1:** The bat samples collected and results of RT-PCR testing for Khosta-1 and Khosta-2 viruses.

Location	Bat Species	Number of Samples Collected		Khosta-1 Virus-Positive Samples (% *)		Khosta-2 Virus-Positive Samples (% *)	
		Oral Swabs	Feces	Oral Swabs	Feces	Oral Swabs	Feces
Basement of the building at Research Institute of Medical Primatology (43°26′06.3″ N 39°59′26.4″ E)	Lesser horseshoe bat (*R. hipposideros*)	27	24	0	0	1 (3.7%)	2 (8.3%)
	Mediterranean horseshoe bat (*R. euryale*)	1	1	0	0	0	0
Museinaya cave (43°33′34.3″ N 39°53′46.2″ E)	Greater horseshoe bat (*R. ferrumequinum*)	4	2	0	0	0	0
	Lesser horseshoe bat (*R. hipposideros*)	3	2	0	0	0	0
Khosta 1 cave (43°33′49.5″ N 39°53′57.2″ E)	Greater horseshoe bat (*R. ferrumequinum*)	21	13	0	1 (7.7%)	0	0
	Common bent-wing bat *Miniopterus schreibersii*	3	1	0	0	0	0
Kolokolnaya cave (43°33′08.3″ N, 39°56′02.4″ E)	Greater horseshoe bat (*R. ferrumequinum*)	36	24	4 (11%)	15 (62.5%)	0	0
	Mediterranean horseshoe bat (*R. euryale*)	2	0	0	0	0	0
Partizanskaya cave (43°37′38.86″ N, 39°54′46.06″ E)	Greater horseshoe bat (*R. ferrumequinum*)	2	1	0	0	0	0
	Lesser horseshoe bat (*R. hipposideros*)	5	3	0	1 (33%)	0	0
Attic of house (44°0′57.51″ N, 39°15′3.63″ E)	Lesser horseshoe bat (*R. hipposideros*)	6	4	0	0	0	0
Krasnoaleksandrovskaya cave (44°0′57.21″ N, 39°21′49.68″ E)	Greater horseshoe bat (*R. ferrumequinum*)	1	0	0	0	0	0
	Lesser horseshoe bat (*R. hipposideros*)	6	0	0	0	0	0
	Myotis bat *Myotis* spp.	3	0	0	0	0	0
Attic of house, Izmaylovka village (43°37′51.72″ N 39°49′45.38″ E)	Lesser horseshoe bat (*R. hipposideros*)	0	2	0	0	0	0
Total		120	77	4 (4.6% **)	17 (14.9% **)	1 (0.89% **)	2 (1.75% **)

* The percentages quoted are indicative and not statistically reliable; ** values calculated only for horseshoe bats, common bent-wing bat, and myotis bat were excluded from the calculation.

**Table 2:** Identity (%) of deduced amino-acid sequences of proteins of Khosta-1 and Khosta-2 viruses with certain representatives of the *Sarbecovirus* subgenus (lineage B of betacoronaviruses).

Protein	Viruses	Amino-Acid Identity (%)								
		Bat SARS-CoV-like BGR/2008 (Bulgaria, 2008)	Bat SARS-CoV-like BtKY72 (Kenya, 2007)	Bat SARS-CoV-like (China, 2005–2016) *	Civet SARS-CoV-like SZ3 (China, 2003)	SARS-CoV Urbani (2003)	Bat SARS-CoV-2-like RaTG13 (China, 2013)	Pangolin SARS-CoV-2-like (China, 2017)	SARS-CoV-2 Wuhan-Hu-1 (2019)	Khosta-1 vs. Khosta-2
ORF1a	Khosta-1	92.95	84.6	81.53–81.6	81.67	81.53	77.2	77.89	77.32	82
	Khosta-2	81.1	80.9	79.4–79.6	79.5	79.4	76.3	77.1	76.45	
ORF1b	Khosta-1	99.07	96.3	95.82–96.3	96.15	96.15	94.22	94.22	94.21	94.75
	Khosta-2	94.7	93.7	94.9–95.17	95.02	94.9	93.44	93.47	93.5	
S	Khosta-1	84.37	89.11	75.5–76.2	75.7	75.7	73.0	72.4	72.22	82
	Khosta-2	79.54	79.7	73.03–73.9	73.2	73.0	72.5	71.74	72.54	
S RBD	Khosta-1	81.3	90.0	77.1–78.5	78.0	76.7	74.0	74.2	72.2	80
	Khosta-2	74.9	80.0	64.3–75.4	75.3	75.9	67.5	68.5	69.0	
ORF3	Khosta-1	85.98	86.7	66.8–72.3	70.8	70.8	65.1	66.2	64.7	81.8
	Khosta-2	77.9	82.22	67.9–69.34	67.15	67.5	64.5	65.8	63.27	
E	Khosta-1	89.47	98.7	87.0	87	87	93.42	93.42	93.42	94.7
	Khosta-2	88.16	94.74	90.7	90.7	90.7	89.5	89.5	89.5	
M	Khosta-1	95.0	97.29	91.86–92.31	92.31	91.86	88.24	87.73	88.13	91
	Khosta-2	90.9	90.5	88.7–89.6	90.5	89.6	87.3	87.27	87.0	
ORF6	Khosta-1	68.25	63.0	49.21–52.38	49.21	49.21	50.82	50.82	50.82	58.73
	Khosta-2	58.1	58.1	44.4–47.6	46.03	46.03	46.7	46.7	46.7	
ORF7a	Khosta-1	69.7	70.6	58–59.7	61.34	61.34	58.5	59.32	58.5	73.5
	Khosta-2	63.25	70.34	58.2–59.26	60.0	60.0	60.0	58.3	59.13	
ORF7b	Khosta-1	86.05	81.4	71.8	71.8	71.8	61.5	71.8	74.4	70.7
	Khosta-2	71.4	73.1	64.2	64.2	64.2	64.2	64.2	64.2	
N	Khosta-1	96.64	92.6	88.36–88.9	89.1	89.1	87.9	87.6	87.4	91.85
	Khosta-2	91.13	90.21	85.75–86.73	86.5	86.5	85.5	86.4	85.24	

* Strains of bat SARS-CoV-like viruses from China included HKU3 (2005; DQ022305), Rs672 (2006; FJ588686), RsSHC014 (2011; KC881005), WIV1 (2012; KF367457), Rs3367 (2012; KC881006), WIV16 (2013; KT444582), and YN2018B (2016; MK211376).

## Data Availability

The genome sequences of BtCoV/Khosta-1/Rh/Russia/2020 and BtCoV/Khosta-2/Rh/Russia/2020 are available in GenBank under accession numbers MZ190137 and MZ190138.

## References

[1] Lau S.K., Woo P.C., Li K.S., Huang Y., Tsoi H.W., Wong B.H., Wong S.S., Leung S.Y., Chan K.H., Yuen K.Y. (2005). Severe acute respiratory syndrome coronavirus-like virus in Chinese horseshoe bats. Proc. Natl. Acad. Sci. USA.

[2] Li W., Shi Z., Yu M., Ren W., Smith C., Epstein J.H., Wang H., Crameri G., Hu Z., Zhang H. (2005). Bats are natural reservoirs of SARS-like coronaviruses. Science.

[3] De Groot R.J., Baker S.C., Baric R., Enjuanes L., Gorbalenya A.E., Holmes K.V., Perlman S., Poon L., Rottier P.J.M., Talbot P.J., King A.M., Adams M.J., Carstens E.B., Lefkowitz E.J. (2012). Family Coronaviridae. Virus Taxonomy: Classification and Nomenclature of Viruses: Ninth Report of the International Committee on Taxonomy of Viruses.

[4] Fan Y., Zhao K., Shi Z.-L., Zhou P. (2019). Bat Coronaviruses in China. Viruses.

[5] Rihtarič D., Hostnik P., Steyer A., Grom J., Toplak I. (2010). Identification of SARS-like coronaviruses in horseshoe bats (Rhinolophus hipposideros) in Slovenia. Arch. Virol..

[6] Ar Gouilh M., Puechmaille S.J., Diancourt L., Vandenbogaert M., Serra-Cobo J., Lopez Roïg M., Brown P., Moutou F., Caro V., Vabret A. (2018). SARS-CoV related Betacoronavirus and diverse Alphacoronavirus members found in western old-world. Virology.

[7] Balboni A., Palladini A., Bogliani G., Battilani M. (2011). Detection of a virus related to betacoronaviruses in Italian greater horseshoe bats. Epidemiol. Infect..

[8] Drexler J.F., Gloza-Rausch F., Glende J., Corman V.M., Muth D., Goettsche M., Seebens A., Niedrig M., Pfefferle S., Yordanov S. (2010). Genomic Characterization of Severe Acute Respiratory Syndrome-Related Coronavirus in European Bats and Classification of Coronaviruses Based on Partial RNA-Dependent RNA Polymerase Gene Sequences. J. Virol..

[9] Wang L.F., Shi Z., Zhang S., Field H., Daszak P., Eaton B.T. (2006). Review of bats and SARS. Emerg. Infect. Dis..

[10] Drexler J.F., Corman V.M., Drosten C. (2014). Ecology, evolution and classification of bat coronaviruses in the aftermath of SARS. Antivir. Res..

[11] Buchfink B., Xie C., Huson D.H. (2014). Fast and sensitive protein alignment using DIAMOND. Nat. Methods.

[12] Tamura K., Peterson D., Peterson N., Stecher G., Nei M., Kumar S. (2011). MEGA5: Molecular evolutionary genetics analysis using maximum likelihood, evolutionary distance, and maximum parsimony methods. Mol. Biol. Evol..

[13] Lole K.S., Bollinger R.C., Paranjape R.S., Gadkari D., Kulkarni S.S., Novak N.G., Ingersoll R., Sheppard H.W., Ray S.C. (1999). Full-length human immunodeficiency virus type 1 genomes from subtype C-infected seroconverters in India, with evidence of intersubtype recombination. J. Virol..

[14] Martin D.P., Murrell B., Golden M., Khoosal A., Muhire B. (2015). RDP4: Detection and analysis of recombination patterns in virus genomes. Virus Evol..

[15] Tong S., Conrardy C., Ruone S., Kuzmin I.V., Guo X., Tao Y., Niezgoda M., Haynes L., Agwanda B., Breiman R.F. (2009). Detection of novel SARS-like and other coronaviruses in bats from Kenya. Emerg. Infect. Dis..

[16] Li F. (2013). Receptor recognition and cross-species infections of SARS coronavirus. Antivir. Res..

[17] Wan Y., Shang J., Graham R., Baric R.S., Li F. (2020). Receptor Recognition by the Novel Coronavirus from Wuhan: An Analysis Based on Decade-Long Structural Studies of SARS Coronavirus. J. Virol..

[18] Andersen K.G., Rambaut A., Lipkin W.I., Holmes E.C., Garry R.F. (2020). The proximal origin of SARS-CoV-2. Nat. Med..

[19] Zhou P., Yang X.L., Wang X.G., Hu B., Zhang L., Zhang W., Si H.R., Zhu Y., Li B., Huang C.L. (2020). A pneumonia outbreak associated with a new coronavirus of probable bat origin. Nature.

[20] Boni M.F., Lemey P., Jiang X., Lam T.T.Y., Perry B.W., Castoe T.A., Rambaut A., Robertson D.L. (2020). Evolutionary origins of the SARS-CoV-2 sarbecovirus lineage responsible for the COVID-19 pandemic. Nat. Microbiol..

[21] Holmes E.C., Goldstein S.A., Rasmussen A.L., Robertson D.L., Crits-Christoph A., Wertheim J.O., Anthony S.J., Barclay W.S., Boni M.F., Doherty P.C. (2021). The origins of SARS-CoV-2: A critical review. Cell.

[22] Kumar N., Kaushik R., Tennakoon C., Uversky V.N., Mishra A., Sood R., Srivastava P., Tripathi M., Zhang K.Y.J., Bhatia S. (2021). Evolutionary Signatures Governing the Codon Usage Bias in Coronaviruses and Their Implications for Viruses Infecting Various Bat Species. Viruses.

[23] Zhou H., Ji J., Chen X., Bi Y., Li J., Wang Q., Hu T., Song H., Zhao R., Chen Y. (2021). Identification of novel bat coronaviruses sheds light on the evolutionary origins of SARS-CoV-2 and related viruses. Cell.

[24] Zhou H., Chen X., Hu T., Li J., Song H., Liu Y., Wang P., Liu D., Yang J., Holmes E.C. (2020). A Novel Bat Coronavirus Closely Related to SARS-CoV-2 Contains Natural Insertions at the S1/S2 Cleavage Site of the Spike Protein. Curr. Biol..

[25] Hu D., Zhu C., Ai L., He T., Wang Y., Ye F., Yang L., Ding C., Zhu X., Lv R. (2018). Genomic characterization and infectivity of a novel SARS-like coronavirus in Chinese bats. Emerg. Microbes Infect..

[26] Wacharapluesadee S., Tan C.W., Maneeorn P., Duengkae P., Zhu F., Joyjinda Y., Kaewpom T., Chia W.N., Ampoot W., Lim B.L. (2021). Evidence for SARS-CoV-2 related coronaviruses circulating in bats and pangolins in Southeast Asia. Nat. Commun..

[27] Delaune D., Hul V., Karlsson E.A., Hassanin A., Ou T.P., Baidaliuk A., Gámbaro F., Prot M., Tu V.T., Chea S. (2021). A novel SARS-CoV-2 related coronavirus in bats from Cambodia. Nat. Commun..

[28] Temmam S., Pasteur I., Vongphayloth K., Salazar E.B., Munier S., Bonomi M. Coronaviruses with a SARS-CoV-2-like receptor-binding domain allowing ACE2-mediated entry into human cells isolated from bats of Indochinese peninsula. https://www.researchsquare.com/article/rs-871965/v1.

[29] Murakami S., Kitamura T., Suzuki J., Sato R., Aoi T., Fujii M., Matsugo H., Kamiki H., Ishida H., Takenaka-Uema A. (2020). Detection and Characterization of Bat Sarbecovirus Phylogenetically Related to SARS-CoV-2, Japan. Emerg. Infect. Dis..

[30] Li W., Moore M.J., Vasllieva N., Sui J., Wong S.K., Berne M.A., Somasundaran M., Sullivan J.L., Luzuriaga K., Greeneugh T.C. (2003). Angiotensin-converting enzyme 2 is a functional receptor for the SARS coronavirus. Nature.

[31] Lan J., Ge J., Yu J., Shan S., Zhou H., Fan S., Zhang Q., Shi X., Wang Q., Zhang L. (2020). Structure of the SARS-CoV-2 spike receptor-binding domain bound to the ACE2 receptor. Nature.

[32] Conceicao C., Thakur N., Human S., Kelly J.T., Logan L., Bialy D., Bhat S., Stevenson-Leggett P., Zagrajek A.K., Hollinghurst P. (2020). The SARS-CoV-2 Spike protein has a broad tropism for mammalian ACE2 proteins. PLoS Biol..

[33] Damas J., Hughes G.M., Keough K.C., Painter C.A., Persky N.S., Corbo M., Hiller M., Koepfli K.P., Pfenning A.R., Zhao H. (2020). Broad host range of SARS-CoV-2 predicted by comparative and structural analysis of ACE2 in vertebrates. Proc. Natl. Acad. Sci. USA.

[34] Ren W., Qu X., Li W., Han Z., Yu M., Zhou P., Zhang S.-Y., Wang L.-F., Deng H., Shi Z. (2008). Difference in Receptor Usage between Severe Acute Respiratory Syndrome (SARS) Coronavirus and SARS-like Coronavirus of Bat Origin. J. Virol..

[35] Ge X.Y., Li J.L., Yang X.L., Chmura A.A., Zhu G., Epstein J.H., Mazet J.K., Hu B., Zhang W., Peng C. (2013). Isolation and characterization of a bat SARS-like coronavirus that uses the ACE2 receptor. Nature.

[36] Hu B., Zeng L.P., Yang X.L., Ge X.Y., Zhang W., Li B., Xie J.Z., Shen X.R., Zhang Y.Z., Wang N. (2017). Discovery of a rich gene pool of bat SARS-related coronaviruses provides new insights into the origin of SARS coronavirus. PLoS Pathog..

[37] Lau S.K.P., Feng Y., Chen H., Luk H.K.H., Yang W.-H., Li K.S.M., Zhang Y.-Z., Huang Y., Song Z.-Z., Chow W.-N. (2015). Severe Acute Respiratory Syndrome (SARS) Coronavirus ORF8 Protein Is Acquired from SARS-Related Coronavirus from Greater Horseshoe Bats through Recombination. J. Virol..

[38] Ge X.Y., Wang N., Zhang W., Hu B., Li B., Zhang Y.Z., Zhou J.H., Luo C.M., Yang X.L., Wu L.J. (2016). Coexistence of multiple coronaviruses in several bat colonies in an abandoned mineshaft. Virol. Sin..

